# The Significance of Psychological Support in Managing Depression in Parkinson's Disease: Combining Venlafaxine with Pramipexole and Psychological Care

**DOI:** 10.62641/aep.v53i1.1663

**Published:** 2025-01-05

**Authors:** Zhiping Huang, Dandan Xiao, Yumei Lao, Xinxin Lai, Wenyu Huang, Decong Zhou

**Affiliations:** ^1^Geriatrics Department, Geriatric Hospital of Hainan, 571100 Haikou, Hainan, China; ^2^Physical Examination Center, Geriatric Hospital of Hainan, 571100 Haikou, Hainan, China; ^3^Quality Control Management Department, Geriatric Hospital of Hainan, 571100 Haikou, Hainan, China; ^4^Neurology Department, Geriatric Hospital of Hainan, 571100 Haikou, Hainan, China

**Keywords:** psychological intervention, venlafaxine, pramipexole, depression, Parkinson's disease

## Abstract

**Background:**

Depression is a common comorbidity in patients with Parkinson's disease (PD) and can significantly impact their overall well-being. The combination of venlafaxine and pramipexole is a standard treatment approach for depression in PD. However, the effects of incorporating psychological care into the treatment regimen remain unclear. This study aimed to investigate the impact of psychological intervention in the treatment of depression in Parkinson’s disease, using a combination of venlafaxine and pramipexole.

**Methods:**

The clinical data of 151 patients with both Parkinson's disease (PD) and depression, treated in Geriatric Hospital of Hainan from May 2021 to May 2023, were analyzed retrospectively. Among the 151 patients, 71 received routine nursing care and were allocated to the control group, while the remaining 80 patients received psychological nursing care based on routine nursing care and were assigned to the study group. The Hamilton Depression Rating Scale (HAMD) and the Hamilton Anxiety Scale (HAMA) were used to evaluate the degree of depression and anxiety in both groups before and after care. The MOS 36-Item Short-Form Health Survey (SF-36) was employed to assess the quality of life of both groups before and after care. The efficacy and adverse reactions in both groups were also analyzed.

**Results:**

Before care, the HAMD and HAMA scores did not significantly differ between the two groups (*p* > 0.05). However, after care, both groups exhibited a significant reduction in HAMD and HAMA scores (*p* < 0.0001), with a more pronounced decrease observed in the study group (*p* < 0.0001). Prior to care, there was no significant difference in SF-36 scores between the two groups (*p* > 0.05). However, following care, the SF-36 scores markedly increased in both groups (*p* < 0.0001), with a more pronounced increase in the study group (*p* < 0.0001). Additionally, a significantly lower overall response rate was noted in the control group compared to the study group (*p* = 0.013), while no significant difference was observed in the total incidence of adverse reactions between the two groups (*p* = 0.273).

**Conclusion:**

Utilizing venlafaxine combined with pramipexole in the treatment of depression in PD, supplemented by psychological nursing care, significantly enhances therapeutic efficacy. This combined approach effectively alleviates symptoms of depression and anxiety in patients without introducing additional side effects. Hence, it emerges as a valuable clinical treatment option.

## Introduction

Parkinson’s disease (PD) is a neurological disorder that primarily disrupts normal motor function, often manifesting with symptoms such as tremors, stiffness, and abnormal posture [1]. Concurrently, individuals with PD frequently experience depression, which significantly impacts their physical and mental well-being, disrupting their daily lives and, in severe cases, leading to suicidal ideation [2, 3]. Moreover, those afflicted with both PD and depression are vulnerable to sleep disturbances and cognitive impairments, posing considerable challenges in clinical management [4].

Patients with both PD and depression are commonly managed with medication [5]. Venlafaxine, a modern antidepressant renowned for its potent anti-anxiety and antidepressant properties, exerts rapid therapeutic effects, thereby enhancing patient adherence [6]. Pramipexole, a non-ergot dopamine receptor agonist, enhances dopamine receptor activity in the striatum and substantia nigra, effectively alleviating clinical symptoms in patients with PD and depression [7, 8]. Nonetheless, research indicates that medication alone may not address the underlying issues comprehensively, necessitating a systematic psychological assessment for optimal outcomes [9]. Therefore, further investigation into psychological interventions alongside drug combination therapy for depression in Parkinson’s patients is warranted.

Therefore, this study investigates the impact of psychological care on the treatment of depression in PD when using venlafaxine combined with pramipexole. The aim is to offer valuable insights for future therapeutic approaches and nursing strategies in managing depression in PD.

## Methods and Data

### Sample Information

The clinical data of 180 PD patients with depression, treated in Geriatric Hospital of Hainan from May 2021 to May 2023, were retrospectively analyzed.

### Inclusion and Exclusion Criteria

Inclusion criteria: Individuals meeting the diagnostic criteria of PD in *China’s Guidelines for the Treatment of Parkinson’s Disease *(Fourth Edition) and the diagnostic criteria of depression in the 4th edition of the *American Diagnostic and Statistical Manual of Mental Disorders* [10, 11]; patients who took venlafaxine and pramipexole under the guidance of doctors of other hospitals; patients who were able to complete the scale evaluation; patients with required clinical records.

Exclusion criteria: Individuals with other Parkinson’s syndrome or parkinsonism-plus syndromes; patients with depression secondary to organic encephalopathy or certain drugs; patients suffering from malignant tumor; patients with a history of severe mental disorder; patients with serious organic diseases such as heart, brain and liver; patients who dropped out from the study halfway.

### Sample Screening 

Following the outlined criteria, 180 patients were screened, and ultimately 151 patients who met the research requirements were selected for the study. Among these, 71 patients who received routine care were assigned to the control group, while the remaining 80 patients, who received additional psychological care alongside routine care, were allocated to the study group.

### Methods

Each patient in both groups received treatment with venlafaxine and pramipexole as per the instructions provided by physicians from other hospitals. Venlafaxine hydrochloride sustained-release tablets (Chengdu Kanghong Pharmaceutical Group Co., Ltd., Chengdu, China; State Food and Drug Administration (SFDA) approval no.: H20070269; Specification: 75 mg/tablet) were administered, starting at an initial dose of 75 mg once daily, with dosage adjustments made gradually based on the patient’s condition. The maximum dose allowed was 225 mg. Additionally, patients were prescribed pramipexole dihydrochloride tablets (CSPC OUYI Pharmaceutical Co., Ltd., Shijiazhuang, China, SFDA approval no.: H20193413; Specification: 0.25 mg/tablet), starting at an initial dose of 0.375 mg three times daily, with dosage adjustments made gradually in accordance with the patient’s condition. The maximum dose permitted was 1.5 mg. Both medications were administered continuously for a duration of 8 weeks.

The control group received standard nursing care, which encompassed admission guidance, dietary guidance, monitoring of vital signs, observation of illness progression, adherence to physician recommendations, and prevention measures for bedsores.

The study group was given psychological nursing care on the basis of routine care measures: (1) Personalized psychological counseling was implemented, with nursing staff instructed to utilize a range of psychological treatment methods, including active listening, counseling, and offering support and assurance. Staff members were tasked with delivering tailored health education and informative guidance to each patient, considering factors such as the patient’s disease condition, age, and educational background. The aim was to foster a comprehensive, accurate, and objective understanding of the patient’s own illness, including its causes, treatment methods, and prognosis. Additionally, staff members were encouraged to share relevant successful case studies to inspire patient motivation for treatment and offer positive psychological reinforcement [12]. (2) Group-based mutual psychological assistance was facilitated through organized group communication activities involving 8–10 patients per group. Patients were encouraged to openly share their emotional struggles and experiences, fostering a supportive environment to alleviate psychological pressures and negative emotions. Nursing staff actively participated in coordinating these group activities, guiding positive discussions and assisting patients in cultivating proactive thinking throughout the process. (3) Mobilizing the involvement of family members was emphasized in the treatment process. Prior to formulating a therapeutic plan, doctors engaged in active communication with the patient’s family, relatives, and friends to gather pertinent information about the patient’s background, work history, lifestyle, and other relevant details. This comprehensive approach allowed for a deeper understanding of the patient’s condition. Furthermore, doctors analyzed the gathered information thoroughly to gain insights into the underlying causes of depression and other symptoms. Additionally, doctors maintained ongoing communication with the patient’s family and relatives, providing regular updates on the patient’s condition and encouraging family members to visit and support the patient frequently. It was recommended that family members offer words of encouragement and engage in supportive actions. Regular telephone visiting hours were also established to facilitate communication between patients and their relatives and friends outside the hospital. (4) Music and exercise therapy: Music therapy has been identified as an effective approach in preventing and alleviating depressive symptoms, serving as a treatment modality for depression [13]. Nursing staff were tasked with organizing daily sessions for patients to listen to melodious and calming music as part of the treatment plan aimed at alleviating depression symptoms in PD. Additionally, patients were encouraged to engage in Taijiquan exercises daily to enhance posture balance and muscle relaxation.

### Observation Index

Primary outcome measures: (1) Depression and anxiety degree: The Hamilton Depression Rating Scale (HAMD) was employed to evaluate depression in both groups before and after care [14]. This scale consists of a total score of 54 points, with higher scores indicating more severe depression. Additionally, the Hamilton Anxiety Scale (HAMA) was utilized to assess anxiety levels in both groups before and after care [15]. The HAMA scale has a total score of 56 points, with higher scores indicating greater severity of anxiety. (2) Efficacy: The effectiveness of interventions in both groups was assessed using the following criteria: The HAMD scores of each patient before and after care were compared. If the reduction rate in HAMD score before and after care exceeded 75%, the treatment was classified as markedly effective. If the reduction rate in HAMD score before and after care was between 25% and 75%, the therapy was considered effective. Treatment was deemed ineffective if the reduction rate in HAMD score was below 25%. The overall response rate was calculated as follows: (Number of markedly effective treatments + Number of effective treatments)/the sum of cases × 100%.

Secondary outcome measures: (1) Quality of life (QoL): The patients’ QoL was evaluated using the MOS 36-Item Short-Form Health Survey (SF-36) before and after nursing [16]. This survey yields a total score of 100 points, with higher scores reflecting better QoL. Additionally, adverse reactions were analyzed in both groups, including gastrointestinal reactions, headaches, dizziness, dysarteriotony, and constipation.

### Statistical Analyses

The collected data were statistically processed using SPSS v20.0 (SPSS Inc., Chicago, IL, USA). GraphPad 8 software package (GraphPad Software Inc., San Diego, CA, USA) was utilized for visualizing the data in the required figures. For measurement data, a normality test was conducted. Data with a normal distribution were presented as mean ± standard deviation (SD). Inter-group and intra-group comparisons were conducted using the independent-samples *t*-test and the paired *t*-test, respectively. Counting data were described as percentages (%) and analyzed using the chi-square test, with results presented as χ^2^. A significance level of *p* < 0.05 was considered statistically significant.

## Results

### Baseline Data

Inter-group comparison of baseline data indicated no significant difference in terms of age, gender, history of smoking, etc. (*p* > 0.05, Table 1).

**Table 1.** **Baseline data**.

		Study group (n = 80)	Control group (n = 71)	χ ^2^	*p* value
Age				0.288	0.592
	≥60 years old	35	28		
	<60 years old	45	43		
Gender				1.741	0.187
	Male	48	35		
	Female	32	36		
BMI				3.307	0.069
	≥23 kg/m^2^	31	38		
	<23 kg/m^2^	49	33		
Course of disease				0.171	0.680
	≥5 years	25	20		
	<5 years	55	51		
History of smoking				1.883	0.170
	Yes	31	20		
	No	49	51		
History of alcoholism				0.194	0.660
	Yes	24	19		
	No	56	52		
Place of residence				1.443	0.230
	Rural areas	58	45		
	Urban areas	22	26		

BMI, Body mass index.

### Comparison of Depression and Anxiety

**Fig. 1.**
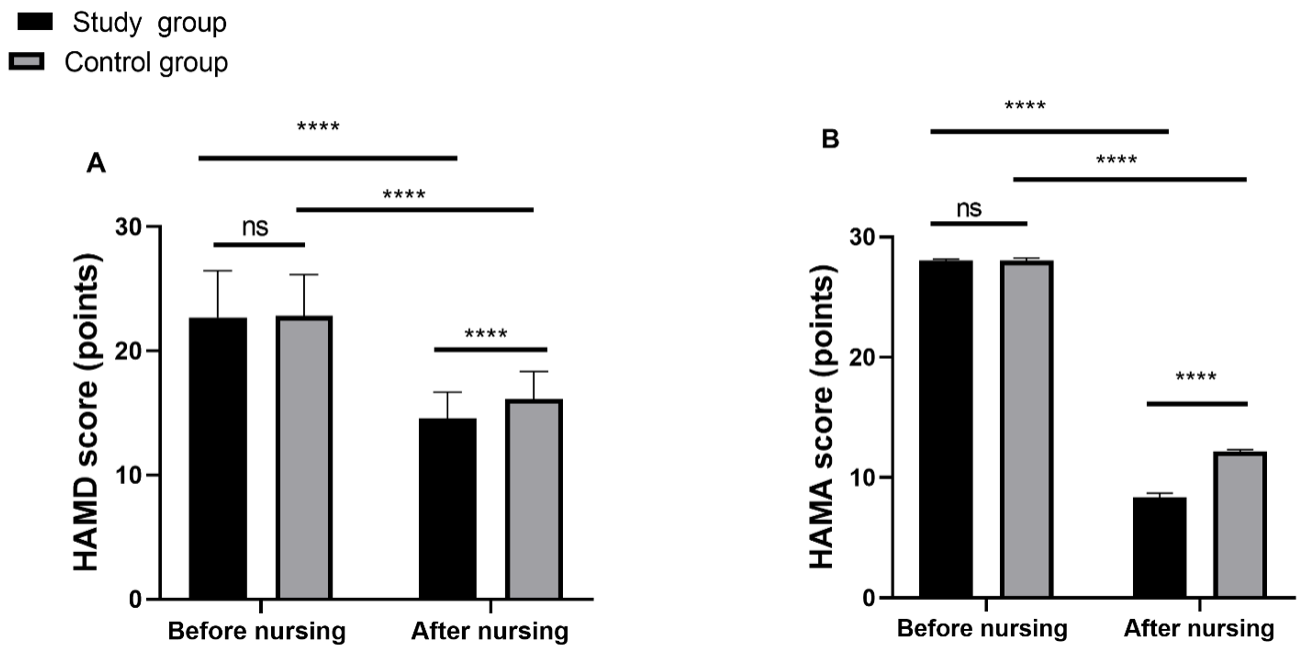
**Depression and anxiety degree of the two groups before and after nursing**. (A) Inter-group comparison of HAMD scores before and after nursing; (B) Inter-group comparison of HAMA scores before and after nursing; Notes: ns, non-significant; *p* > 0.05; **** *p* < 0.0001. HAMD, Hamilton Depression Rating Scale; HAMA, Hamilton Anxiety Scale.

Before treatment, there were no significant differences in HAMD and HAMA scores between the two groups (*p* > 0.05). However, following treatment, both groups experienced significant reductions in HAMD and HAMA scores (*p* < 0.0001). Furthermore, the study group exhibited significantly lower HAMD and HAMA scores compared to the control group (*p* < 0.0001, Fig. 1).

### Comparison of QoL 

**Fig. 2.**
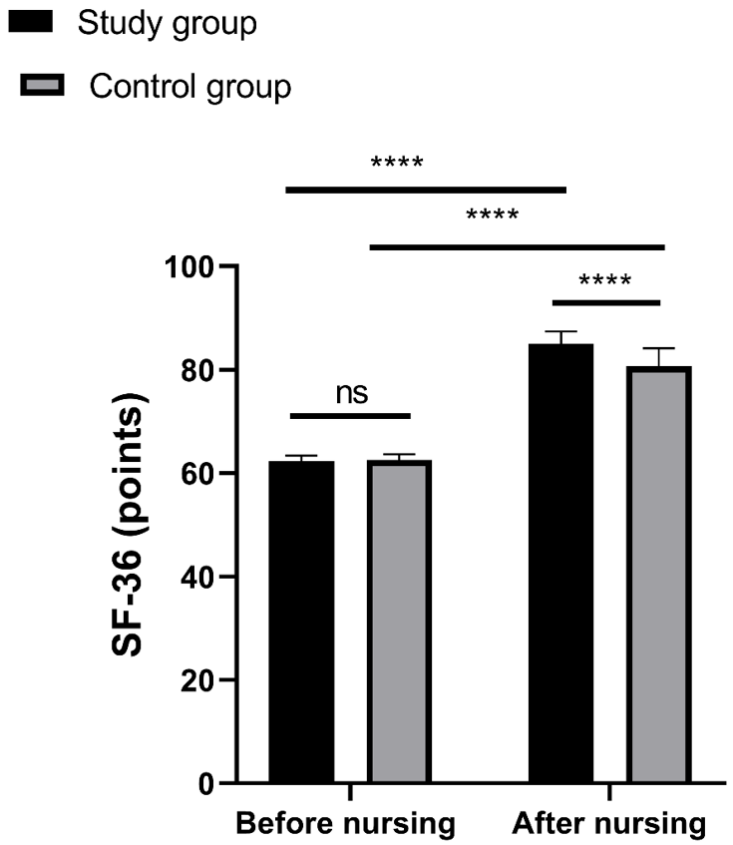
**SF-36 scores of the two groups before and after nursing**. Notes: ns, non-significant; *p* > 0.05; **** *p* < 0.0001. SF-36, MOS 36-Item Short-Form Health Survey.

Before treatment, there were no significant inter-group differences found in SF-36 scores (*p* > 0.05). However, following treatment, the SF-36 scores of both groups notably increased (*p* < 0.0001), with a more pronounced increase observed in the study group (*p* < 0.0001, Fig. 2).

### Comparison of Efficacy

Inter-group comparison of clinical efficacy revealed a significantly lower overall response rate in the control group compared to the study group (*p* = 0.013, Table 2).

**Table 2.** **Comparison of efficacy between two groups [n (%)]**.

Group	Markedly effective	Effective	Ineffective	Overall response
Study group (n = 80)	43 (53.75)	32 (40.00)	5 (6.25)	75 (93.75)
Control group (n = 71)	25 (35.21)	32 (45.07)	14 (19.72)	57 (80.28)
χ ^2^	5.223	0.396	6.203	6.203
*p* value	0.022	0.529	0.013	0.013

### Comparison of Adverse Reactions

Statistical analysis of adverse reactions showed no significant difference between the control and study groups regarding the total incidence of adverse reactions (*p* = 0.273, Table 3).

**Table 3.** **Incidence of adverse reactions [n (%)]**.

Group	Gastrointestinal reactions	Headache and dizziness	Dysarteriotony	Constipation	Total adverse reaction
Study group (n = 80)	1 (1.25)	2 (2.50)	1 (1.25)	1 (1.25)	5 (6.25)
Control group (n = 71)	1 (1.41)	4 (5.63)	1 (1.41)	2 (2.82)	8 (11.27)
χ ^2^	0.007	0.968	0.007	0.474	1.204
*p* value	0.932	0.325	0.932	0.491	0.273

## Discussion

PD stands as one of the most prevalent neurodegenerative conditions. Afflicted individuals often experience depressive symptoms, characterized by persistent feelings of sadness, sleep disturbances, loss of interest in daily activities, and a general sense of negativity and pessimism [17, 18, 19]. These symptoms not only diminish patients’ quality of life but also hasten the progression of motor symptoms [20]. Venlafaxine, a selective serotonin and norepinephrine reuptake inhibitor, rapidly alleviates depression by augmenting the levels of these neurotransmitters [21]. Pramipexole, acting as a non-ergot dopamine receptor agonist, targets D2 and D3 receptors in the dopamine system, directly addressing depression in PD, particularly with its high affinity for D3 receptors [22]. The combined use of venlafaxine and pramipexole offers a synergistic effect in alleviating depressive symptoms. Nevertheless, individuals coping with PD and depression endure considerable physical and psychological distress, significantly impacting their overall well-being and quality of life [23]. Consequently, it becomes imperative to actively incorporate psychotherapeutic interventions into their treatment regimen. This study delves into the impact of psychological care in the management of depression in PD, particularly in conjunction with venlafaxine and pramipexole therapy.

In this study, both groups experienced significant reductions in HAMD and HAMA scores after treatment, with more pronounced decreases observed in the study group. These findings suggest that psychological nursing, when combined with venlafaxine and pramipexole therapy, can effectively alleviate depression and anxiety in PD patients compared to routine nursing care alone. Furthermore, following treatment, the study group exhibited notably higher SF-36 scores than the control group, indicating that psychological nursing can enhance patients’ quality of life more effectively in conjunction with medication therapy. Additionally, the study revealed a significantly lower overall response rate in the control group compared to the study group, with no notable difference in the total incidence of adverse reactions between the two groups. These results suggest that psychological nursing care enhances treatment efficacy without increasing adverse events. Several factors contribute to these outcomes, including individualized psychological nursing measures, effective counseling, group-based mutual psychological assistance, and active involvement of patients’ families in the care process. Moreover, supplemental music and exercise therapy further augment the overall antidepressant treatment effect [24]. As Rosa Quelhas [25] has emphasized, the treatment of PD and its associated mental symptoms should be personalized and include psychotherapeutic interventions, aligning with the conclusions drawn from this study.

The study has affirmed the beneficial effects of psychological nursing care in the treatment of depression in PD when combined with venlafaxine and pramipexole, as evidenced by retrospective analysis. However, it is essential to acknowledge certain limitations. Firstly, the retrospective nature of the study and the limited sample size may introduce inherent biases. Secondly, the study did not explore the long-term prognosis of patients, highlighting the need for further investigation into the impact of psychological nursing care on the long-term outcomes of PD patients treated with venlafaxine combined with pramipexole. Therefore, future studies should aim for a more comprehensive and thorough analysis to evaluate the efficacy of psychological nursing care in treating PD patients. By addressing these limitations, we can aspire to obtain more robust and effective experimental results, thereby offering valuable insights into the application of psychological nursing care for PD patients.

## Conclusion

In conclusion, psychological nursing care, when combined with venlafaxine and pramipexole therapy for depression in PD, significantly enhances treatment efficacy. This combined approach effectively alleviates symptoms of depression and anxiety in patients without introducing additional adverse reactions, thus representing a valuable option for clinical use.

## Availability of Data and Materials

All data generated or analyzed during this study are included in this published article.
